# Challenges to the Israeli healthcare system: attracting medical students to primary care and to the periphery

**DOI:** 10.1186/s13584-018-0218-z

**Published:** 2018-05-29

**Authors:** Charles Weissman, Rachel Yaffa Zisk-Rony, Alexander Avidan, Uriel Elchalal, Howard Tandeter

**Affiliations:** 1 Department of Anesthesiology and Critical Care Medicine, Hadassah-Hebrew University Medical Center, Hebrew University – Hadassah School of Medicine, Kiryat Hadassah, POB 12000, 91120 Jerusalem, Israel; 2 Hadassah Henrietta Szold School of Nursing, Hebrew University, Jerusalem, Israel; 3 Department of Obstetrics and Gynecology, Hadassah-Hebrew University Medical Center, Hebrew University Hadassah School of Medicine, Jerusalem, Israel; 4 Department of Family Medicine, Ben Gurion University Joyce and Irving Goldman School of Medicine, Be’er Sheva, Israel

**Keywords:** Medical students, Medical education, Residency, Medical specialty selection, Career choice

## Abstract

**Background:**

The greatest challenges facing healthcare systems include ensuring a sufficient supply of primary care physicians and physicians willing to work in rural or peripheral areas. Especially challenging is enticing young physicians to practice primary care in rural/peripheral areas. Identifying medical students interested in primary care and in residencies in Israel’s periphery should aid the healthcare leadership. It may be particularly important to do so during the clinical years, as this is the stage at which many future physicians begin to crystallize their specialty and location preferences.

**Methods:**

Questionnaires, distributed to 6 consecutive 5th-year classes of the Hebrew University – Hadassah School of Medicine, from 2010 to 2016, elicited information on criteria for choosing a career specialty, criteria for choosing a residency program and whether one-time monetary grants authorized in the 2011 physicians’ union contract would attract students to residencies in the periphery.

**Results:**

Completed questionnaires were returned by 511 of 740 (69%) students. Ninety-eight (19%) were interested in a primary care residency, 184 (36%) were unsure and 229 (45%) were not interested. Students interested in primary care were significantly less interested in specialties that perform procedures/surgeries and in joining a medical school faculty, while being more inclined towards specialties dealing with social problems, controllable lifestyles and working limited hours. The percentage of students interested in primary care was stable during the study period.

Forty-eight of the students indicated interest in residencies in the country’s periphery, and 42% of them were also interested in primary care residencies. Overall, only 3.7% of students were interested in both a primary care residency and a residency in the periphery.

Thirty percent of the students indicated that the monetary incentives tempted them to consider a residency in the periphery. Fifty-three percent of these students reported that they did not yet know the geographic area where they wished to do their residency, as compared to only 22% among those not interested in incentives.

**Conclusions:**

This study provides the healthcare leadership with information on the characteristics of the students at a centrally-located medical school who tend to be more interested in primary care and in working in the periphery. Specifically, the study found that students interested in primary care desire a positive life/work balance, something that Israeli non-hospital primary care practice provides. Students considering residencies in the periphery were similarly inclined. Moreover, about a third of students had positive thoughts about monetary incentives for residencies in peripheral hospitals. These students should be identified early during their clinical experience so that attempts to recruit them to the periphery can commence before their specialty and location preferences have fully crystallized. Parallel studies should be performed at additional Israeli medical schools.

## Background

The greatest challenges facing healthcare systems include providing sufficient numbers of primary care and rural physicians [1]. Especially challenging is enticing young physicians to establish primary care practices in rural areas [1, 2]. Among the counties where such challenges exist are the United States, Australia and Canada [1, 3, 4]. Israel suffers from a similar problem with shortages of family medicine specialists in peripheral areas of the country. The latter include the northern and southern regions of the country which encompass the majority of the county’s rural districts. These areas chronically suffer from physician shortages, greater infant mortality and lower life expectancy than the rest of the country [5]. In an attempt to remedy this maldistribution, the physicians’ union contract of 2011 included both pay increases for practicing in the periphery and one-time monetary incentives for moving and committing to work in the periphery [6, 7]. This programs also provided incentives for medical students interested in residencies in specialties suffering workforce shortages. The original program excluded family medicine residents from both the incentives for work in the periphery and the incentives to work in distressed specialties. However, in 2015 the Israel Ministry of Health began to provide financial incentives to family medicine residents willing to train in peripheral areas.

The Israeli healthcare system is dependent on primary care physicians to provide comprehensive out-patient care and to serve along with community specialists and internal Health Maintenance Organization regulations and pre-authorization systems as gatekeepers for secondary and tertiary care. These primary care physicians, family medicine specialists, pediatricians, internists and general practitioners, are based in health maintenance organization clinics. Despite family medicine and pediatrics being popular specialties among Israeli medical students, there is still a nationwide shortage of family medicine specialists and pediatricians, especially in the county’s peripheral areas. This shortage is predicted to increase as the population grows, ages, and life spans lengthen. Furthermore, the primary care physician population is aging as many physicians who emigrated from the former Soviet Union in the 1990’s are reaching retirement age [8]. Therefore, it is necessary to attract more medical students to primary care residencies. To increase recruitment it is important that the medical education and healthcare system leadership better understand the characteristics of students interested in pursuing primary care and how they differ from those without such interests. The leadership needs similar information on students interested in residency positions in the county’s periphery.

The present study compared the characteristics of Israeli 5th-year (out of 6 years) medical students interested in primary care residencies and residencies in peripheral areas with those without such interests. The dataset used was collected over a 6-year period from a single Israeli medical school and includes over 500 students. The two hypotheses tested were that the student interest in primary care would be greater among women medical students and that interest in residencies in peripheral areas would be greater among students who attended high school in the northern and southern regions. We also explored whether the one-time monetary grants approved in the 2011 physicians’ union contract would encourage students to consider a residency in a peripheral hospital. The ultimate goal was to provide the medical education and healthcare leaderships with the attributes that typify students interested in primary care and/or rural practice. Fifth-year students were studied since our previous study revealed that most had already begun the process of deciding on a specialty [9]. In order for the healthcare leadership to influence specialty decisions, it is important to be cognizant of the thought patterns of the students early in their decision process.

## Methods

This study included students from 6 consecutive 5th-year classes of the Hebrew University – Hadassah School of Medicine in Jerusalem (2010–2016). It utilized a questionnaire to examine various aspects of the medical specialty selection process. The questionnaire was based on the results of factor analysis from a questionnaire used previously [9]. This permitted us to reduce repeat Likert scale questions thus providing space for new ones that investigated additional issues. Among the new topics examined were the influence of family and colleagues on specialty and residency program decisions as well as the interests of the students in academic pursuits. The questionnaire included multiple choice questions, free-text queries and 5-point Likert scales. In addition to demographic information, the questionnaire elicited information about: (1) Whether the student had already considered a specialty for their residency, which specialty or specialties they were considering (free-text), when they had first considered a specialty and whether and when (prior to beginning medical school or when during the first 5 years of medical school) they had changed their mind; (2) The criteria for choosing a career specialty {20 items, 10 new, 5-point Likert scale}; (3) The criteria for choosing a residency program {20 items, 9 new, 5-point Likert scale}; and (4) The importance of interest in a specific specialty when choosing a residency {3 new items, multiple choice}. (5) Whether the one-time monetary grants authorized in the 2011 physicians’ union contract would attract them to a residency in a peripheral hospital (1 new item, final 4 classes).

After two small (15 students) preliminary studies designed to identify problems and test the questionnaire’s user-friendliness, the questionnaires were distributed to the 5th year classes of the Hebrew University – Hadassah School of Medicine in Jerusalem during the 2010–2011, 2011–2012, 2012–2013, 2013–2014, 2014–2015 and 2015–2016 school-years. A parallel article examining medical student subgroups also utilized this dataset [10].

### Data analysis

Data were entered into Microsoft Excel (Redmond, WA) spreadsheets and analyses were performed with Systat 12 (San Jose CA).

#### Primary care

The dataset was divided into three groups based on the answer to the 5-point Likert scale question: “Are you interested in a primary care residency?” Group 1 included the two points representing positive tendencies; Group 2 included the neutral point; and Group 3 the two points representing negative tendencies. This permitted us to compare students interested and not interested in a primary care residency, while also examining those who were unsure. The results from each of the 6 school-years were compared to determine whether there were differences between years.

#### Rural (periphery) workforce

Initial data analysis showed that a significant number of students interested in primary care were interested in a residency in the county’s periphery. Therefore, a *post-hoc* examination was made of the characteristics of students interested in a residency in the country’s periphery. The dataset was divided into two groups based on the answer to the 5-point Likert scale question: “Are you interested in a residency in the country’s periphery?” Group A included the two points representing positive tendencies while Group B included the two points representing negative tendencies.

#### Incentives

The data set was divided into three groups as per the responses to the multiple- choice query “As the result of the union contract of 2011, residents in peripheral hospitals receive a one-time monetary incentive and higher salaries: (1) These incentives attract me to a residency in the periphery (2) I already plan to do a residency in the periphery (3) The incentives do not attract me to a residency in the periphery”. The differences between the characteristics of the three groups were determined.

Based on prior research demonstrating significant gender differences associated with specialty selection^,^ an a priori decision was made to separately analyze and compare the male and female data [11].

Responses to multiple choice questions are presented as frequency distributions. When the Likert Scale results were considered continuous variables, statistical analyses were performed using all 5 points. When presented as categorical variables the Likert Scale results were compressed into three categories, (the two points representing negative tendencies and the two points representing positive tendencies were each combined). The percentages of total responses for each of the three categories (positive tendency, middle point and negative tendency) were then computed.

For continuous data, differences between the groups were analyzed using analysis of variance with Tukey post-hoc tests. Categorical data were analyzed using χ^2^ or Fisher exact tests, as appropriate. A *p* value < 0.05 was assumed to represent statistical significance. Univariate linear regression analysis was used to examine the association between the answers to two queries. Backward multivariate and logistic regression analyses were performed with the dependent variable being either interest in a residency in primary care or a peripheral hospital. The independent variables were the demographic parameters and specialty and resident selection criteria.

Criteria for specialty and residency program selection were subjected to factor analysis (principal components analysis) using varimax rotation with set eigenvalues of ≥1.0. The data were also analyzed with hierarchal cluster analysis.

The Institutional Review Board of the Hadassah Medical Organization approved this study. Completion of the questionnaire by the student was considered tacit consent.

## Results

Completed questionnaires were returned by 511 of 740 (69%) 5th-year medical students.

### Primary care

Ninety-eight (19%) students were interested in pursuing a primary care residency, 184 (36%) were unsure and 229 (45%) were not interested. Demographic information is found in Table 1. Interest in primary care among 5th-year students was stable over the 6-year study period ranging from 17%–21% annually.

**Table 1:** Primary care - demographic and other information

		Primary care	Undecided	No primary care	Primary care vs no primary care	Primary care		Females vs males
						Females	Males	
N		98	184	229		41	57	
Female		42%	51%	49%				
Male		58%	50%	51%	NS			
Age (years)	18–20	0%	0%	0.4%		0%	0%	
	21–23	25%	14%	17%		27%	23%	
	24–26	19%	31%	29%		34%	9%	
	27–29	37%	42%	39%		27%	44%	
	30–32	14%	10%	12%		10%	18%	
	+ 32	5%	4%	3%	NS	2%	7%	*p* < 0.01
Marital status	Single	70%	63%	73%		66%	72%	
	Married	30%	36%	26. %		34%	26%	
	Divorced	1%	1%	0.4%	NS	0%	2%	NS
Thought of a specialty when started	Yes	85%	79%	82%		85%	84%	
	No	15%	21%	18%	NS	15%	16%	NS
When did you start thinking of a specialty?	Pre - med school	25%	29%	26%		27%	23%	
	Year 1	4%	4%	4%		6%	2%	
	Year 2	4%	4%	2%		3%	4%	
	Year 3	4%	3%	5%		0%	6%	
	Year 4	56%	42%	48%		62%	51%	
	Year 5	9%	19%	16%	NS	3%	13%	NS
Have you changed your mind?	Yes	61%	59%	63%		59%	62%	
	No	40%	41%	38%	NS	41%	38%	NS
When did you change your mind?	Year 1	0%	0%	2%		0%	0%	
	Year 2	0%	3%	1%		0%	0%	
	Year 3	2%	1%	2%		0%	4%	
	Year 4	41%	44%	42%		35%	46%	
	Year 5	57%	52%	54%	NS	65%	50%	NS
Specialties under consideration	Family medicine	16%	2%	0%		14%	17%	
	Pediatrics	41%	42%	24%		54%	30%	
	Internal med	26%	30%	32%		26%	26%	
	Ob/Gyn	19%	20%	22%		34%	7%	
	Emergency med	4%	0%	2%		0%	7%	
	Surgical*	20%	31%	31%		9%	30%	
	Other	22%	44%	39%	*p* < 0.01	20%	30%	*p* < 0.01
High school location	Israel	97%	97%	96%		95%	98%	
	Other	3%	3%	4%	NS	5%	2%	NS
High school location in israel	Jerusalem	32%	25%	23%		26%	36%	
	Central	29%	37%	44%		31%	29%	
	North	26%	28%	23%		33%	21%	
	South	13%	9%	10%		10%	14%	
	Other	0%	1%	1%	NS	0%	0%	NS
Future residency location	Israel	98%	98%	99%		97%	98%	
	Other	2%	2%	1%	NS	3%	2%	NS
Perferred residency location in Israel	Jerusalem	28%	18%	23%		23%	31%	
	Central	22%	32%	34%		13%	29%	
	North	17%	11%	9%		23%	12%	
	South	1%	1%	2%		3%	0%	
	Don’t know	33%	39%	32%	*p* < 0.05	39%	29%	*p* < 0.01

*Surgical specialties

Compared to students not interested in primary care, those interested in primary care were significantly less interested in a specialty with procedures/surgeries and becoming “members of a medical school faculty”, while being more inclined towards a specialty dealing with social problems (Tables 2 and 3). When choosing a residency program, students attracted to primary care were more interested than their colleagues in a residency in the country’s periphery. Alternately, 42% of the 48 students who indicated interest in a residency program in the country’s periphery were also interested in a primary care residency (Table 2). When asked about specialties they were considering, students interested in primary care expressed significantly more interest in family medicine and pediatrics than those not interested in primary care. Among the latter, none were considering family medicine (Table 1).

**Table 2:** Primary care - selection criteria

	Primary care	Undecided	No primary care	Primary care vs no primary	Primary care		Females vs males
					Females	Males	
N	98	184	229		41	57	98
Criteria for choosing a specialty							
Time with family (1)	**76%**	73%	65%	*p* < 0.03	**85%**	68%	*p* < 0.05
Specialty with team work	**57%**	54%	45%	*p* < 0.04	56%	58%	NS
Influence of spouse	**43%**	43%	31%	*p* < 0.007	**49%**	39%	*p* < 0.05
Specialty that deals with social issues (3)	**42%**	35%	22%	*p* < 0.001	**51%**	35%	*p* < 0.009
Daytime work only (1)	**26%**	29%	16%	*p* < 0.03	**34%**	19%	*p* < 0.03
Work only in the community	**9%**	3%	4%	*p* < 0.001	12%	7%	NS
Procedures/surgery	43%	41%	**57%**	*p* < 0.01	34%	**49%**	*p* < 0.02
High salary	36%	48%	**51%**	*p* < 0.002	34%	38%	NS
Opportunity for research (2)	34%	37%	**44%**	*p* < 0.05	27%	39%	NS
Academic faculty member	19%	26%	**30%**	*p* < 0.05	24%	16%	NS
Criteria for choosing a residency							
Much supervision by senior physicians	**53%**	45%	37%	*p* < 0.001	**68%**	42%	*p* < 0.04
Limited work hours	**29%**	30%	17%	*p* < 0.001	33%	27%	NS
Short residency (<4.5. years)	**28%**	15%	12%	*p* < 0.001	29%	26%	NS
Much clinic time (2)	**23%**	16%	5%	*p* < 0.001	20%	25%	NS
Hospital in the periphery (3)	**19%**	7%	6%	*p* < 0.001	20%	19%	NS
Leading department (1)^a^	67%	77%	**79%**	*p* < 0.03	71%	65%	NS
Large hospitial	52%	55%	**62%**	*p* < 0.04	59%	47%	NS
Family living location	78%	75%	64%	NS	**88%**	70%	*p* < 0.05
Making clinical decisions on your own	66%	50%	55%	NS	56%	**74%**	*p* < 0.05
Pre-determined work hours (2)	47%	48%	35%	NS	**59%**	39%	*p* < 0.05
Influence of family	42%	37%	31%	NS	**54%**	33%	*p* < 0.01
Many on-call shifts	10%	11%	12%	NS	5%	**14%**	*p* < 0.03

Percent of "agree" and "agree much" responses on 5-point Likert Scale Numbers in parenthesis are the results of factor analysis

^a^clusters per cluster analysis

Bold result indicates the higher value in a statistically significant pair

**Table 3:** Regression analysis

Demographics/criteria	Multivariable Backward regression coefficient		Backward logistic regression		
		*p*	Odds ratio	95% confidence limits	*P*
Interest in a primary care residency	*r* = 0.43				
Male gender	0.264	0.015	2.251	1.195–4.241	0.012
Criteria for choosing a specialty					
Opportunity for research	−0.096	0.033	0.755	0.578–0.987	0.04
High salary	−0.207	0.002	0.580	0.376–0.893	0.013
Influency of spouse	0.122	0.018			
Specialty that deals with social issues	0.146	0.004	1.442	1.079–1.927	0.013
Work only in the community	0.278	0.001			
Wide range of medical problems			1.512	1.004–2.277	0.048
Criteria for choosing a residency program	*r* = 0.45				
Leading department	−0.125	0.053	0.581	0.395–0.854	0.006
Influence of family	0.094	0.026	1.414	1.083–1.847	0.011
Short residency (< 4.5 years)	0.159	0.001	1.524	1.122–2.069	0.007
Peripheral hospital	0.188	0.001			
Much supervision by senior physicians	0.202	0.001	1.836	1.340–2.515	0.001
Much clinic time	0.207	0.001	1.495	1.110–2.013	0.008
Interest in a residency in a peripheral hospital	r = 0.43				
Older age	0.157	0.004	1.732	1.144–2.621	0.009
Have considered a specialty	−0.234	0.048			
High school location (periphery)	0.138	0.002	1.546	1.089–2.193	0.015
Criteria for choosing a specialty					
Wide range of medical problems	0.144	0.008			
Time with family	0.145	0.009	1.686	1.001–2.839	0.049
Work only in the community	0.178	0.002	1.701	1.147–2.524	0.008
Influence of spouse			1.488	1.019–2.173	0.039
Private practice	−0.109	0.012			
Specialty that deals with social issues	0.123	0.003			
Narrow range of medical problems	0.298	0.001	2.156	1.377–3.376	0.001
Criteria for choosing a residency program	*r* = 0.52				
Specific location in Israel	−0.106	0.031			
Leading department	−0.280	0.001	0.480	0.091–0.791	0.004
Limited work hours	0.108	0.031			
Family living location	0.115	0.046			
Many on-call shifts	0.121	0.011			
Teaching students	0.144	0.001	1.563	1.061–2.304	0.024
Physical challenge	0.157	0.001			
Primary care	0.168	0.002			
Much clinic time	0.198	0.003	1.893	1.288–2.783	0.001
Much supervision by senior physicians			0.045	1.010–2.455	0.045
Influence of family			1.430	1.030–1.986	0.022
Monetary incentives for residency in the periphery					
	r - 0.58				
Opportunity for research	−0.126	0.047	0.580	0.360–0.933	0.025
Specialty advancing rapidly	−0.143	0.022	0.620	0.393–0.90	0.041
Specific location	−0.204	0.001	0.428	0.275–0.667	0.001
Primary care	0.115	0.023			
Specialty that deals with social issues	0.126	0.008	1.530	1.044–2.242	0.029
Peripheral hospital	0.258	0.001	2.835	1.782–4.511	0.001

As can be seen in Tables 1 and 2 (Appendix A), there was similar interest in a primary care residency between female (17%) and male (21%) students. Comparisons between men and women students interested in primary care showed that women rated lifestyle issues, such as family time, more highly than men and were more interested in pediatrics (Tables 1 and 2, Appendix A).

### Rural (periphery) workforce

Differences between students expressing and not interested in a residency in the country’s periphery are found in Table 4 (Appendix B). Results of multivariate and logistic regression analyses are in Table 3.

**Table 4:** Residency in a peripheral hospital

		Periphery	No periphery	Periphery vs no Periphery
N		48	382	
Gender	Female	45%	48%	
	Male	55%	52%	NS
Age (years)	18–20	0%	0.3%	
	21–23	17%	18%	
	24–26	19%	30%	
	27–29	42%	39%	
	30–32	17%	10%	
	+ 32	6%	3%	NS
Marital status	Single	63%	71%	
	Married	38%	29%	
	Divorced	0%	1%	NS
High school location	Israel	100%	97%	
	Other	0%	3%	NS
High school location in Israel	Jerusalem	17%	27%	
	Central	31%	42%	
	North	38%	22%	
	South	15%	8%	
	Other	0%	1%	*p* < 0.05
Residency location	Israel	100%	98%	
	Other	0%	2%	NS
Future residency location in Israel	Jerusalem	13%	26%	
	Central	16%	36%	
	North	40%	7%	
	South	13%	1%	
	Don’t know	18%	31%	*p* < 0.01
Specialties under consideration	Family medicne	0%	2%	
	Pediatrics	46%	30%	
	Internal medicine	24%	32%	
	Ob/Gyn	15%	21%	
	Emergency medicine	5%	1%	
	Surgical specialties	32%	30%	
	Other specialties	32%	35%	*p* < 0.05
Criteria for choosing a specialty				
Time with family	85%		66%	*p* < 0.001
Controllable lifestyle	75%		65%	*p* < 0.04
Influency of spouse	56%		37%	*p* < 0.01
Specialty that deals with social issues	46%		27%	*p* < 0.004
Work only in the community	19%		3%	*p* < 0.001
Narrow range of medical problems	10%		2%	*p* < 0.001
Advancing rapidly	48%		62%	*p* < 0.05
Opportunity for research	29%		41%	*p* < 0.03
Criteria for choosing a residency program				
Family living location	81%		69%	*p* < 0.04
Teaching students	57%		40%	*p* < 0.05
Pre-determined work hours	56%		41%	*p* < 0.05
Influence of family	52%		33%	*p* < 0.02
Much supervision bysenior physicians	50%		42%	*p* < 0.03
Primary care	42%		15%	*p* < 0.0004
Limited work hours	42%		21%	*p* < 0.03
Much clinic time	33%		8%	*p* < 0.001
Leading department	58%		79%	*p* < 0.0001
Large hosptial	47%		60%	*p* < 0.003

Percent of “agree” and “agree much” responses on 5-point Likert Scale

Interactions between the replies to the questions “Are you interested in a residency in the country’s periphery?” and “Are you interested in a primary care residency?” revealed that 4% of all the students were interested in both a primary care residency and a residency in the periphery (Appendix C).

#### Incentives

Responses to the query about monetary incentives for a residency in peripheral hospitals are in Tables 5 (Appendix D). Thirty percent of the students reported that the incentives interested them, while another 6% had already decided to do a residency in the periphery. The relationships between the responses to this question and those to the query “are you interested in a residency in the country’s periphery?” revealed that 82% of the students who replied they were not attracted by the incentives indicated that they were not interested in a residency in the periphery while those that reported that the incentives interested them showed less aversion (20% negative tendency and 54% positive tendency) to a residency in the periphery.

**Table 5:** Incentives - residency in periphery

		1. Incentive interests me	2. Plan peripheral residency	3. Incentives don’t interest me	1 vs 3	1 vs 2	2 vs 3
N		106	20	223			
Percent		30%	6%	64%			
Gender	Female	48%	47%	50%			
	Male	52%	53%	50%	NS	NS	NS
Age (years)	**18–20**	0%	0%	0%			
	**21–23**	22%	20%	19%			
	**24–26**	27%	15%	31%			
	**27–29**	36%	50%	35%			
	**30–32**	10%	10%	11%			
	**+ 32**	5%	5%	3%	NS	NS	*p* < 0.02
Marital status	Single	68%	60%	72%			
	Married	30%	40%	27%			
	Divorced	2%	0%	1%	NS	*p*< 0.05	*p* < 0.02
High school location in Israel	Jerusalem	27%	5%	25%			
	Central	36%	32%	40%			
	South	10%	26%	10%			
	Other	1%	0%	1%	NS	*p* < 0.01	NS
Future residency location in Israel	Jerusalem	14%	10%	29%			
	Central	20%	0%	41%			
	North	14%	55%	8%			
	South	0%	20%	1%			
	Don’t know	53%	15%	22%	*p*< 0.03	*p* < 0.01	*p*< 0.01
Specialties under consideration	Pediatrics	39%	31%	31%			
	Internal medicine	21%	6%	35%			
	Ob/Gyn	29%	19%	15%			
	Emergency medicine	0%	13%	1%			
	Surgical specialties	32%	31%	29%			
	Other specialties	31%	31%	37%	*p* < 0.04	*p* < 0.01	*p* < 0.02
Criteria for choosing a specialty							
Time with family (1)		**78%**	60%	63%	*p* < 0.004	*p* < 0.04	NS
Specialty that deals with social issues (3)		**35%**	35%	25%	*p* < 0.02	NS	NS
Advancing rapidly (2)		51%	45%	**69%**	*p* < 0.0007	NS	*p* < 0.04
Opportunity for research (2)		29%	5%	**47%**	*p* < 0.0001	*p* < 0.04	*p* < 0.0001
Controllable lifestyle (1)		74%	50%	66%	NS	NS	NS
Independent practice		54%	40%	51%	NS	*p* < 0.03	*p* < 0.03
High salary		51%	20%	45%	NS	*p* < 0.006	*p* < 0.01
Procedures/surgery		45%	32%	51%	NS	NS	*p* < 0.04
Private practice		37%	10%	404%	NS	*p* < 0.008	*p* < 0.009
Criteria for choosing a residency							
Controllable lifestyle		**73%**	45%	58%	*p* < 0.04	*p* < 0.03	NS
Primary care		**27%**	32%	15%	*p* < 0.001	NS	*p* < 0.002
Hospital in the periphery (3)		**8%**	55%	4%	*p* < 0.001	*p* < 0.0007	*p* < 0.001
Intellectual challenge (1)^a^		8%	60%	**87%**	*p* < 0.004	NS	*p* < 0.007
Leading department (1)^a^		69%	45%	**85%**	*p* < 0.0002	*p* < 0.004	*p* < 0.0009
Specific location		51%	68%	**70%**	*p* < 0.0001	NS	NS
Large hospitial		50%	26%	**68%**	*p* < 0.005	*p* < 0.04	*p* < 0.0006
Opportunity for research		17%	5%	**29%**	*p* < 0.02	*p* < 0.01	*p* < 0.0005
Much supervision by senior physicians		44%	20%	46%	NS	NS	*p* < 0.04

Percent of “agree” and “agree much” responses on 5-point Likert Scale

Numbers in parenthesis are the results of factor analysis

^a^Clusters per cluster analysis

Bold result indicares the higher value in a statistically significant pair

## Discussion

The present study identified several medical student characteristics associated with interest in a primary care residency and those interested in a residency in the periphery among 5th year students at the Hadassah-Hebrew University Medical School.

### Primary care

There were many differences between 5th-year Israeli medical students interested and not interested in a primary care residency. Students inclined toward primary care were more interested in lifestyle: spending time with their families, working limited hours and working only during the daytime. This importance of lifestyle was more pronounced in female than male students. Students inclined toward primary care were less interested in academic pursuits, such as being academic faculty members. Reduced interest in academic activities was also observed among Japanese medical students with preferences for family medicine [12]. Lack of interest in academic endeavors is problematic since it reduces the number of family medicine faculty members able to serve as medical student mentors. This lack of mentors might decrease the ability to attract students to the specialty.

It is important to note that the query on the questionnaire was about the broader area of primary care and not specifically about family medicine. Unlike a previous study where we found a female-predominance among 6th year Israeli medical students interested in family medicine, in the present study we did not find such predominance [13]. Furthermore, the proportion of women medical students interested and not interested in primary care was comparable. Similarly, upon multiple regression analysis, interest in primary care was not associated with being female. We thus failed to prove our hypothesis that interest in primary care would be greater among women than men medical students. This variance with our previous studies is attributable to primary care incorporating general internal medicine, general pediatrics and some aspects of obstetrics/gynecology, in addition to family medicine. We previously found that In Israel, internal medicine and obstetrics/gynecology residencies attract many male students [13]. When asked which specialties they were considering, students interested in primary care listed pediatrics and internal medicine more frequently than family medicine.

In many countries, attracting medical students to primary care careers is a daunting task [14]. The reasons for this difficulty differ between countries [15]. In the United States, the proportion of medical students selecting primary care specialties dropped from 73% in 1996 to 44% in 2008, although subsequently there has been some stabilization [16]. Moreover, more internal medicine and pediatric residents are choosing to subspecialize, reducing the numbers entering general internal medicine and pediatric practices [17]. The major reasons cited for the dearth of students entering primary care in the United States are relatively low incomes in the face of high student debt burdens, many administrative tasks and time pressures [16]. Many medical schools have instituted programs to attract more students to primary care, with a multi-year exposure to primary care being more successful than adding a single primary care course to a conventional curriculum [18]. Other countries face similar problems. In Vietnam less than a third of commune (collective farming communities) health stations are staffed by a physician even though the number of medical school graduates almost tripled between 2004 and 2011 [19]. The reasons include poor working conditions, low income and lack of opportunities for career development [19]. In a survey of 9499 South Korean medical students only 2.2% expressed interest in family medicine [20].

Shortages of primary care physicians are generally attributed to low salaries, lack of prestige and glamor; long hours with frequent on-call responsibilities; and lack of a controllable lifestyle [21, 22]. The situation in Israel differs from other countries in that primary care physicians mainly work in health maintenance clinics, receive salaries comparable to other physicians, have few on-call obligations and have set hours [8]. This was reflected in our previous study where Israeli 6th-year students rated family medicine and pediatrics as specialties with controllable lifestyles and positive relationships between controllable lifestyle and remuneration [13]. This was also found in the present study, where compared to 5th-year students not interested in primary care, those interested in primary care wanted a specialty with time for family involving only daytime work and practice in the community (i.e. outside the hospital). This interest profile was similarly demonstrated by their greater interest in short (in years) residency programs with limited hours and with much time spent in clinics. This grouping of interests indicates a desire for positive life/work balance, something that Israeli non-hospital primary care practice provides. A recent study of Israeli family medicine residents reported similar findings. Specifically, more than 85% of residents reported that factors that positively influence their choice included the ability to combine work, family, and free time; direct, meaningful contact with patients; the diversity of patients and medical conditions; and attractive working conditions [23]. This interest profile is similar to those reported from other countries among students interested in primary care and family medicine [12, 24]. However, primary care in isolated Israeli rural village has been reported to lead to unclear boundaries between private life and physician roles leading to problems with life/work balance [25]. This may be among the reasons for the shortage of primary care practitioners in the country’s periphery.

### Rural (periphery) workforce

Worldwide, rural areas often suffer physician shortages. Therefore, in many countries with large rural areas, such as the United States, Canada and Australia, emphasis has been placed on encouraging more medical students to become rural primary care physicians [26, 27]. To attract students to rural areas, medical schools have programs that expose students to rural practice and have increased the recruitment of students from rural areas [28]. The current study showed that of the more than 500 5th-year Israeli students studied, 8.9% were considering residency in the country’s periphery. This percentage is greater than that reported in our previous study of 5th year students (4.6%) and might be attributable to the recent introduction of monetary incentives (one-time grants and salary increases) for physicians choosing to train and practice in the periphery. Shortages of rural physicians frequently includes a lack of primary care physicians; a situation also present in Israel. Among students interested in primary care, 19% would choose a residency in the periphery. Alternately, among students considering a residency in the periphery, 42% were interested in primary care. This attraction to primary care among students interested in living in rural areas was also observed among Japanese medical students [29]. However, when we examined our overall student sample, only 3.7% of the 5th-year students reported interest in both primary care and peripheral residencies.

Regression analysis showed that attending high school in Israel’s south and north was associated with interest in residency in the same regions. Notably, significantly more students interested in residencies in the periphery reported that the locale of their family was an important criterion for choosing a residency program. Furthermore, among students who responded to the question concerning the effects of one-time monetary incentives to do a residency in the periphery, “I already plan to do a residency in the periphery”, 63% had gone to high school in the northern or southern areas. Therefore, we confirmed the hypothesis that interest in residencies in peripheral areas is greater among students who attended high school in peripheral regions. Similar observations were made in Kenya, United States, Japan and Australia where students of rural origin were more interested in rural practice [2, 11, 28, 29]. These results have potential healthcare policy implications. Firstly, they can contribute to decisions about whether to admit more medical school applicants residing in the periphery in an attempt to reduce the shortage of physicians practicing there. Secondly, the results can also contribute to decisions about whether to include rural medicine rotations during the clinical years to provide all students with exposure to such medical practice [30].

The characteristics of students considering a residency in the periphery were similar to those interested in a primary care residency. Although this may be partially attributable to the many students interested in a primary care residency, it also likely reflects that those interested in residencies in the periphery are more interested in life-work balance and are less interested in a residency in a large hospital and in a leading department.

### Incentives

The last four years of the study provided an opportunity to explore the student’s thoughts about the monetary incentives for rural residency included in the 2011 union contract [7]. Although only 6% were already planning to do a residency in the periphery, 30% indicated that the incentives induced them to consider a residency there. Fifty-three percent of the latter students reported that they did not yet know where they wished to do their residency as opposed to 22% among those not interested in the incentives. Moreover, there were more differences between the two groups. Students interested in the incentives were more interested in primary care residencies, specialties dealing with social issues, specialties providing family time and residencies affording controllable lifestyles. Alternately, they were less interested in research opportunities, a rapidly advancing specialty and a residency in a leading department in a large hospital. For medical educators and healthcare leaders these results point to a group with distinct characteristics who might be encouraged to join residency programs in the periphery. The challenge is to better characterize this student group, identify them early during their specialty/residency program decision process and provide positive information and counseling about residency programs and lifestyle in the periphery. Thirty percent of the residents working in Israel’s periphery reported that the incentives had influenced them greatly, even though initially they had intended to work there [23]. Previous studies have shown that Israeli primary care practitioners in the periphery are more satisfied and had a broader scope of practice than urban practitioners [29, 31, 32]. While the aim is to market residency programs in the periphery to the students, the results of this study show that a third of the students reported that the influence of family was an important part of their residency selection process. Therefore, consideration should be given to including spouses in recruitment efforts.

### Strengths and limitations

The strength of this study is that the large number of students studied allowed us to examine subgroups, such as those interested in primary care. A further strength was its multi-year design showing that the proportion of students interested in primary care and peripheral hospitals remained steady throughout the study.

The major limitation is that the study was performed in a single institution that is located in the center of the country and is focused on academic medicine. Hence the findings are not automatically generalizable to the entire Israeli medical student population, and parallel studies should be carried out at additional Israeli medical schools.^1^

Another limitation is that the study only included Israeli medical students when half the medical internship workforce is comprised of Israeli who graduated from foreign medical schools and immigrants and 58% of the family medicine residents graduated from such schools [23]. Yet, unlike the foreign graduates who only enter the Israeli healthcare system as interns, having had exposure to the various specialties in foreign healthcare systems during medical school, the Israeli medical students are part of the Israeli healthcare system while they are medical students providing the Israeli healthcare leadership the opportunity to directly expose them to Israeli primary and rural practices early in their clinical experience.

A further limitation is that there might be selection criteria that were not included in the questionnaire. However, both the factor and cluster analyses showed few factors and clusters indicating that a wide-variety of topics were queried.

## Conclusions

The characteristics of students showing interest in primary care and practice in peripheral areas, that were delineated by this study, should aid department heads and residency program directors in identifying potential residents. Moreover, the present study revealed that for Israeli medical students the 5th-year is an important juncture in their choice of a medical specialty. Eighty percent had already considered various specialties. Although, about a quarter had begun their considerations before beginning medical school, the majority had begun during their 4th and 5th-years. Furthermore, 60% of those who had begun the thought process had already changed their minds. Therefore, the 4th and 5th years of medical school appear to be an opportune time to market the various specialties to medical students and might also be the time to begin informing them about residency programs. Since the students’ decisions as to specialty and residency program decisions have major influences on the composition and geographic distribution of the future physician workforce, it is for the healthcare leadership to take the initiative and provide the students with direction, counseling and information to help them with their choices.

## Appendix A

**Table 6:** Primary care - selection criteria

	Primary care	Undecided	No primary care	Primary care vs no primary	Primary care		Females vs males
					Females	Males	
N	98	184	229		41		
Criteria for choosing a specialty							
Time with family (1)	**75.51%**	72.68%	65.07%	*p* < 0.03	**85.37%**	68.42%	*p* < 0.05
Specialty with team work	**57.14%**	53.80%	44.98%	*p* < 0.04	56.10%	57.89%	NS
Influence of spouse	**42.55%**	42.62%	31.14%	*p* < 0.007	**48.65%**	38.60%	p < 0.05
Specialty that deals with social issues (3)	**41.84%**	34.62%	21.93%	*p* < 0.001	**51.22%**	35.09%	*p* < 0.009
Daytime work only (1)	**25.51%**	28.73%	16.23%	p < 0.03	**34.15%**	19.30%	*p* < 0.03
Work only in the community	**9.18%**	2.73%	3.51%	p < 0.001	12.20%	7.02%	NS
Procedures/surgery	42.86%	40.98%	**56.58%**	*p* < 0.01	34.15%	**49.12%**	*p* < 0.02
High salary	36.08%	48.09%	**51.09%**	*p* < 0.002	34.15%	37.50%	NS
Opportunity for research (2)	33.67%	37.16%	**43.67%**	p < 0.05	26.83%	38.60%	NS
Academic faculty member	19.39%	25.68%	**30.26%**	p < 0.05	24.39%	15.79%	NS
Bedside specialty	93.88%	92.93%	92.07%	NS	92.68%	94.74%	NS
Wide range of medical problems	79.38%	71.04%	68.12%	NS	75.61%	82.14%	NS
Controllable lifestyle (1)	69.39%	73.91%	64.19%	NS	78.05%	63.16%	NS
Advancing rapidly (2)	56.12%	61.41%	58.77%	NS	51.22%	59.65%	NS
Independent practice	51.02%	60.11%	52.40%	NS	56.10%	47.37%	NS
Private practice	39.80%	36.96%	42.48%	NS	41.46%	38.60%	NS
Influence of family	12.37%	9.78%	8.81%	NS	15.00%	10.53%	NS
Specialty that my coleagues choose^a^	2.06%	1.64%	0.00%	NS	0.00%	3.57%	NS
Influence of classmates^a^	2.06%	1.63%	0.44%	NS	0.00%	3.57%	NS
Narrow range of medical problems^a^	2.04%	3.26%	3.49%	NS	0.00%	3.51%	NS
Criteria for choosing a residency							
Much supervision by senior physicians	**53.06%**	45.11%	36.68%	*p* < 0.001	**68.29%**	42.11%	*p* < 0.04
Limited work hours	**29.17%**	29.89%	16.67%	*p* < 0.001	32.50%	26.79%	NS
Short residency (< 4.5. Years)	**27.55%**	15.30%	11.79%	*p* < 0.001	29.27%	26.32%	NS
Much clinic time (2)	**22.45%**	16.30%	4.82%	*p* < 0.001	19.51%	24.56%	NS
Hospital in the periphery (3)	**19.39%**	6.52%	6.11%	*p* < 0.001	19.51%	19.30%	NS
Leading department (1)^a^	67.35%	77.17%	**79.04%**	*p* < 0.03	70.73%	64.91%	NS
Large hospitial	52.04%	54.64%	**61.84%**	*p* < 0.04	58.54%	47.37%	NS
Intellectual challenge (1)^a^	83.67%	82.07%	83.41%	NS	87.80%	80.70%	NS
Family living location	77.55%	74.86%	63.76%	NS	**87.80%**	70.18%	*p* < 0.05
Controllable lifestyle	69.39%	70.49%	60.70%	NS	73.17%	66.67%	NS
Making clinical decisions on your own	66.33%	49.46%	55.02%	NS	56.10%	**73.68%**	*p* < 0.05
Specific location in Israel	65.31%	64.48%	63.88%	NS	68.29%	63.16%	NS
Much “action”	52.04%	33.15%	46.49%	NS	51.22%	52.63%	NS
Pre-determined work hours (2)	46.94%	48.37%	35.37%	NS	**58.54%**	38.60%	*p* < 0.05
Physical challenge	47.96%	36.96%	46.93%	NS	41.46%	52.63%	NS
Teaching students	43.88%	43.48%	43.42%	NS	39.02%	47.37%	NS
Influence of family	41.84%	37.16%	31.00%	NS	**53.66%**	33.33%	*p* < 0.01
Opportunity for research	20.41%	25.00%	27.95%	NS	19.51%	21.05%	NS
Many on-call shifts	10.20%	11.41%	11.79%	NS	4.88%	**14.04%**	*p* < 0.03

Percent of “agree” and “agree much” responses on 5-point Likert Scale

Numbers in parenthesis are the results of factor analysis

^a^Clusters per cluster analysis

Bold results representthe higher value of a statistically significant pair

## Appendix B

**Table 7:** Residency in a peripheral hospital

	Periphery	No periphery	Periphery vs no periphery
N	48	382	
Criteria for choosing a specialty			
Time with family	**85.42%**	66.23%	*p* < 0.001
Controllable lifestyle	**75.00%**	65.01%	*p* < 0.04
Influency of spouse	**56.25%**	37.40%	*p* < 0.01
Specialty that deals with social issues	**45.83%**	27.03%	*p* < 0.004
Work only in the community	**18.75%**	2.62%	*p* < 0.001
Narrow range of medical problems	**10.42%**	1.83%	*p* < 0.001
Advancing rapidly	47.92%	**62.30%**	*p* < 0.05
Opportunity for research	29.17%	**40.94%**	*p* < 0.03
Bedside specialty	91.67%	93.10%	NS
Wide rangeof medical problems	62.50%	72.44%	NS
Independent practice	60.42%	55.24%	NS
Specialty with teamwork	54.17%	47.52%	NS
Procedures/surgery	50.00%	47.24%	NS
High salary	50.00%	47.64%	NS
Private practice	47.92%	41.05%	NS
Daytime work only	27.08%	21.78%	NS
Influence of family	14.89%	10.76%	NS
Academic faculty member	18.75%	28.61%	NS
Influence of classmate	4.17%	1.31%	NS
Specialty that my coleagues choose	2.08%	0.79%	NS
Criteria for choosing a residency			
Family living location	**81.25%**	68.59%	*p* < 0.04
Teaching students	**56.25%**	40.05%	*p* < 0.05
Pre-determined work hours	**56.25%**	40.73%	*p* < 0.05
Influence of family	**52.08%**	32.98%	*p* < 0.02
Much supervision by senior physicians	**50.00%**	42.15%	*p* < 0.03
Primary care	**42.22%**	14.72%	*p* < 0.0004
Limited work hours	**41.67%**	21.26%	*p* < 0.03
Much clinic time	**33.33%**	8.38%	*p* < 0.001
Leading department	58.33%	**78.59%**	*p* < 0.0001
Large hosptial	46.81%	**60.05%**	*p* < 0.003
Intellectual challenge	77.08%	82.77%	NS
Controllable lifestyle	77.08%	61.56%	NS
Specific location	75.00%	65.79%	NS
Making clinical decisions on your own	62.50%	53.26%	NS
Much “action”	43.75%	40.31%	NS
Physical challenge	41.67%	41.10%	NS
Short residency	22.92%	15.18%	NS
Opportunity for research	20.83%	25.85%	NS
Many on-call shifts	16.67%	9.14%	NS

Percent of “agree” and “agree much” responses on 5-point Likert Scale

Numbers in parenthesis are the results of factor analysis

^a^Clusters per cluster analysis

Bold results represent the higher value of a statisitcally significant pair

## Appendix C

**Table 8:** In “residency in the periphery” and “primary care residency”

Residency in the periphery					
		Not intersted	Neutral	Interested	Total
Primary	Not interested	37.4%	4.7%	2.7%	44.8%
Care	Neutral	22.7%	11.0%	2.3%	36.0%
Residency	Interested	10.4%	5.1%	**3.7%**	19.2%
	Total	70.5%	20.7%	8.8%	

## Appendix D

**Table 9:** Primary care - selection criteria

	1Incentive interests me	2Plan peripheral residency	3Incentives don’t interest me	1 vs 3	1 vs 2	2 vs 3
N	106	20	223			
Criteria for choosing a specialty						
Time with family (1)	**78.30%**	60.00%	63.39%	*p* < 0.004	*p* < 0.04	NS
Specialty that deals with social issues (3)	**35.24%**	35.00%	25.11%	*p* < 0.02	NS	NS
Advancing rapidly (2)	51.43%	45.00%	**68.75%**	*p* < 0.0007	NS	*p* < 0.04
Opportunity for research (2)	29.25%	5.00%	**46.64%**	*p* < 0.0001	*p* < 0.04	*p* < 0.0001
Bedside specialty	95.28%	95.00%	92.79%	NS	NS	NS
Wide range of medical problems	73.58%	63.16%	73.66%	NS	NS	NS
Controllable lifestyle (1)	73.58%	50.00%	66.07%	NS	NS	NS
Independent practice practice	53.77%	40.00%	51.28%	NS	*p* < 0.03	*p* < 0.03
High salary	50.48%	20.00%	45.09%	NS	*p* < 0.006	*p* < 0.01
Specialty with team work	48.11%	55.00%	50.89%	NS	NS	NS
Procedures/surgery	45.28%	31.58%	50.67%	NS	NS	*p* < 0.04
Influence of spouse	41.35%	45.00%	34.84%	NS	NS	NS
Private practice	36.79%	10.00%	39.64%	NS	*p* < 0.008	*p* < 0.009
Daytime work only (1)	23.30%	21.05%	19.20%	NS	NS	NS
Academic faculty member	22.86%	10.53%	31.25%	NS	NS	NS
Influence of family	9.43%	10.00%	11.21%	NS	NS	NS
Work only in the community	4.72%	10.00%	3.14%	NS	NS	NS
Narrow range of medical problems^a^	4.72%	5.00%	2.68%	NS	NS	NS
Specialty that my coleagues choose^a^	1.90%	5.00%	0.90%	NS	NS	NS
Influence of classmates^a^	0.95%	0.00%	1.34%	NS	NS	NS
Criteria for choosing a residency						
Controllable lifestyle	**73.33%**	45.00%	58.12%	*p* < 0.04	*p* < 0.03	NS
Primary care	**27.18%**	31.58%	15.32%	*p* < 0.001	NS	*p* < 0.002
Hospital in the periphery (3)	**7.55%**	55.00%	3.85%	*p* < 0.001	*p* < 0.0007	*p* < 0.001
Intellectual challenge (1)^a^	80.19%	60.00%	**87.05%**	*p* < 0.004	NS	*p* < 0.007
Leading department (1)^a^	68.87%	45.00%	**84.82%**	*p* < 0.0002	*p* < 0.004	*p* < 0.0009
Specific location	50.94%	68.42%	**69.82%**	*p* < 0.0001	NS	NS
Large hospitial	50.00%	26.32%	**67.71%**	*p* < 0.005	*p* < 0.04	*p* < 0.0006
Opportunity for research	16.98%	5.00%	**29.02%**	*p* < 0.02	*p* < 0.01	*p* < 0.0005
Family living location	68.87%	70.00%	69.06%	NS	NS	NS
Making clinical decisions on your own	49.06%	50.00%	58.04%	NS	NS	NS
Much “action”	45.28%	40.00%	43.75%	NS	NS	NS
Much supervision by senior physicians	43.81%	20.00%	46.43%	NS	NS	*p* < 0.04
Limited work hours	43.40%	30.00%	40.18%	NS	NS	NS
Physical challenge	42.45%	30.00%	41.70%	NS	NS	NS
Teaching students	38.10%	50.00%	48.21%	NS	NS	NS
Influence of family	32.38%	30.00%	34.38%	NS	NS	NSb
Pre-determined work hours (2)	25.71%	25.00%	20.72%	NS	NS	NS
Short residency	15.09%	10.00%	16.52%	NS	NS	NS
Much clinic time (2)	13.33%	20.00%	9.38%	NS	NS	NS
Many on-call shifts	12.26%	10.00%	10.71%	NS	NS	NS

Percent of “agree” and “agree much” responses on 5-point Likert Scale

Numbers in parenthesis are the results of factor analysis

^a^Clusters per cluster analysis

Bold results represent the higher value of a statistically significant pair

## Footnotes

1 Our hypothesis is that the findings for the other medical schools will be similar to what we found for Hebrew University-Hadassah. This is because a) Despite its location in the center of the country, the Hebrew University- Hadassah Medical School draws students from across the country; b) Earlier studies of family medicine specialty preferences found similarities between this medical school and the total for all Israeli medical schools [23].

## References

[1] Allen SM, Ballweg RA, Cosgrove EM, Engle KA, Robinson LR, Rosenblatt RA, Skillman SM, Wenrich MD (2013). Challenges and opportunities in building a sustainable rural primary care workforce in alignment with the Affordable Care Act: the WWAMI program as a case study. Acad Med.

[2] Duffrin C, Diaz S, Cashion M, Watson R, Cummings D, Jackson N (2014). Factors associated with placement of rural primary care physicians in North Carolina. South Med J.

[3] Sempowski IP (2004). Effectiveness of financial incentives in exchange for rural and underserviced area return-of-service commitments: systematic review of the literature. Can J Rural Med.

[4] Del Mar C (2011). New investments in primary care in Australia. BMC Health Serv Res.

[5] Israel Ministry of Health: Disparity in health services and ways of coping with it. 2015, Jerusalem; www.health.gov.il/publicationsfiles/inequality-2015.pdf (Accessed 12 Mar 2017).

[6] Weil LG, Bin Nun G, Mckee M (2013). Recent physician strike in Israel: a health system under stress?. Israel J Health Policy Res.

[7] Simonstein F (2013). Priorities in the Israeli health care system. Med Health Care Philos.

[8] OECD Reviews of Health Care Quality: Israel 2012: Raising Standards OCED Library http://www.oecd-ilibrary.org/social-issues-migration-health/oecd-reviews-of-health-care-quality-israel-2012_9789264029941-en Accessed 26 Feb 2017.

[9] Weissman C, Zisk-Rony RY, Schroeder JE, Weiss YG, Avidan A, Elchalal U, Tandeter H (2012). Medical specialty considerations by medical students early in their clinical experience. Isr J Health Policy Res.

[10] Avidan A, Weissman C, Elchalal U, Tandeter H, Zisk-Rony RY (2018). Medical specialty selection criteria of Israeli medical students early in their clinical experience: subgroups. Isr J Health Policy Res.

[11] Dossajee H, Obonyo N, Ahmed SM (2016). Career preferences of final year medical students as a medical school in Kenya – a cross sectional study. BMC Medical Education.

[12] Kawamoto R, Ninomiya D, Kasai Y, Kusunoki T, Ohtsuka N, Kumagi T, Abe M (2016). Factors associated with the choice of general medicine as a career among Japanese medical students. Med Educ Online.

[13] Weissman C, Tandeter H, Zisk-Rony RY, Weiss YG, Elchalal U, Avidan A (2013). Schroeder JE Israeli medical student's perceptions of six key medical specialties. Israel J Health Policy Res.

[14] Petterson SM, Liaw WR, Tran C, Bazemore AW (2015). Estimating the residency expansion required to avoid projected primary care physician shortages by 2035. Ann Fam Med.

[15] Kringos D, Boerma W, Bourgueil Y, Cartier T, Dedeu T, Hasvold T, Hutchinson A, Lember M, Oleszczyk M, Rotar Pavlic D, Svab I, Tedeschi P, Wilm S, Wilson A, Windak A, Van der Zee J, Groenewegen P (2013). The strength of primary care in Europe: an international comparative study. Br J Gen Pract.

[16] Teng VC, Lin SY (2014). Renewing US medical students’ interest in primary care: bridging the role model gap. Postgrad Med.

[17] Messinger CJ, Hafler J, Khan AM, Long T (2017). Recent trends in primary care interest and career choices among medical students at an academic medical institution. Teach Learn Med.

[18] Pfarrwaller E, Sommer J, Chung C, Maisooeuve H, Nendaz M, Perron NJ, Haller DM (2015). Impact of interventions to increase the proportion of medical students choosing a primary career: a systemic review. J Gen Intern Med.

[19] Giang KB, Minh HV, Hien NV, Ngoc NM, Hinh ND (2017). Knowledge of primary health care and career choice at primary health care settings among final year medical students – challenges to human resources for health in Vietnam. Global Pub Heal.

[20] Kim YY, Kim UN, Kim YS, Lee JS (2016). Factors associated with the specialty choice of Korean medical students: a cross-sectional survey. Hum Resource Health..

[21] Gold JA, Barg FK, Margo K (2014). Undergraduate students’ perspectives on primary care. J Prim Care Comm Health.

[22] Clinite KL, DeZee KJ, Durning SJ, Kogan JR, Blevins T, Chou CL, Diemer G, Dunne DW, Fagan MJ, Hartung PJ, Kazantsev SM, Mechaber HF, Paauw DS, Wong JG, Reddy ST (2014). Lifestyle factors and primary care specialty selection: comparing 2012-2013 graduating and matriculating medical students’ thoughts on specialty lifestyle. Acad Med.

[23] Ashkenazi Y, Gordon M, Yankellevich A, Rosen B (2017). Attracting medical residents to the periphery and to medical specialties in crisis following the 2011 collective agreement.

[24] Kiobassa K, Miksch A, Hermann K, Loh A, Szecsenyi J, Joos S (2011). Becoming a general practitioner – which factors have more impact on career choice of medical students?. BMC Fam Pract.

[25] Monnickendam SM, Borkan JM, Matalon A, Zalewski S (1996). Trials and tribulations of country doctors: a qualitative study of doctor-patient relationships in rural Israel. Isr J Med Sci.

[26] Royston PJ, Mathieson K, Leafman J, Ojan-Sheehan O (2012). Medical student characteristics predictive of intent for rural practice. Rural Remote Health.

[27] Russell DJ, Wakerman J, Humphreys JS (2013). What is a reasonable length of employment for health workers in Australian rural and remote primary healthcare services?. Aust Health Rev.

[28] Puddey IB, Mercer A, Playford DE, Riley GJ (2017). Medical student selection criteria and socio-demographic factors as predictors of ultimately working rurally after graduation. BMC Med Educ.

[29] Kawamoto R, Uemoto A, Ninomiya D, Hasegawa Y, Ohtsuka N, Kusunoki T, Kumagi T, Abe M (2015). Characteristics of Japanese medical students associated with their intention for rural practice. Rural Remote Health.

[30] Williamson MI, Wilson R, McKechnie R, Ross J (2012). Does the positive influence of an undergraduate rural placement persist into postgraduate years?. Rural Remote Health.

[31] Fennig S, Yuval D, Greenstein M, Rabin S, Weingarten M (2000). Job satisfaction among certified and non-certified general practitioners. Isr Med Assoc J.

[32] Biderman A, Shvartzman P, Anson O, Almagor G, Grol R (1996). Responsibility taking and role definition in family practice: effect of training and practice setting. Isr J Med Sci.

